# Linking remote sensing with crop modeling for yield and nitrate leaching predictions in Minnesota

**DOI:** 10.1002/jeq2.70137

**Published:** 2026-01-10

**Authors:** Muhammad Tahir, David J. Mulla

**Affiliations:** ^1^ Department of Soil, Water, and Climate University of Minnesota St. Paul Minnesota USA

## Abstract

Upscaling crop yield and nitrate‐N leaching loss from experimental sites to large areas under alternative crop rotations is crucial for assessing strategies and setting goals to protect groundwater quality at a regional scale. Nitrogen (N) rate field trials were used to calibrate the Environmental Policy Integrated Climate (EPIC) model for continuous‐corn (*Zea mays* L.) (C‐C), corn‐soybean (*Glycine max* L.) (C‐Sb), and alfalfa (*Medicago sativa* L.)‐corn (A‐C), with or without rye (*Secale cereale* L.) cover crop. Satellite estimates of crop evapotranspiration (ET_c_) were used to upscale the EPIC model for crop yield and nitrate‐N leaching, using the irrigation‐water permitting data from 2010 to 2017 for 13,375 ha of sandy soils in Bonanza Valley, central Minnesota. Four alternative management scenarios were evaluated with EPIC: (1) reducing N fertilizer rate from the maximum return to N value (MRTN) (of 0.05 to a value of 0.1 (for the N price/crop value ratio), (2) adding rye cover crop at MRTN of 0.1, (3) irrigating with EPIC auto‐trigger in scenario 2, and (4) converting 50% of C‐C acreage in scenario 3 to A‐C. Nash‐Sutcliffe coefficients, normalized root‐mean‐square error, and *R* ^2^ values based on ET_c_/crop yield for calibration and validation of the EPIC model ranged 0.95–0.54, 4.67–19.4, and 0.96–0.74; and 0.74–0.41, 7.99–23.4, and 0.88–0.55, respectively. Results indicate that corn yield at MRTN of 0.05 averaged 12.5, 13.2, and 13.4 t ha^−1^ under C‐C, C‐Sb, and A‐C rotations, while yields at MRTN of 0.1 were reduced by 4.1%, 3.5%, and 3.3%, respectively. The baseline scenario of C‐C, C‐Sb, and A‐C rotations at MRTN of 0.05 had annual nitrate‐N leaching losses of 51.8, 45.5, and 31.4 kg ha^−1^, while MRTN of 0.1 reduced these losses by 9.1%, 5.0%, and 3.8%, respectively. Rye after corn and soybean reduced nitrate‐N leaching losses in the MRTN of 0.1 scenario by 5.8% and 13.6%, respectively. EPIC auto‐irrigation of corn, soybean, and alfalfa at MRTN of 0.1 reduced nitrate‐N leaching losses with rye (relative to conventional irrigation) by 9.6%, 9.1%, and 8.5%, respectively. Further, replacing half of the C‐C acreage with A‐C rotation would provide a 6.1% reduction, resulting in a total reduction of 27.4% in nitrate‐N leaching to groundwater when all alternative practices are combined. Overall, augmenting EPIC model with field‐observed ancillary data and remote sensing successfully predicted the yield and NO_3_‐N leaching losses under different crop rotations, indicating opportunities to upscale field‐scale agroecosystem simulations, particularly if used to calculate NO_3_‐N leaching on a long‐term basis at the regional scales.

## Core Ideas

- Environmental Policy Integrated Climate (EPIC) model successfully simulated crop yield and NO_3_‐N leaching under alternative management scenarios.
- METRIC‐Earth Engine Evapotranspiration Flux, crop evapotranspiration, and irrigation water estimates were used to upscale the EPIC model predictions from field‐scale to the entire Bonanza Valley.
- The maximum return to N value at 0.1 (N price/crop value ratio), cover crop, and EPIC auto‐trigger irrigation reduced NO_3_‐N leaching in Bonanza Valley by 21.3% from baseline conditions.
- Shifting 50% corn‐corn to alfalfa‐corn in Bonanza Valley can reduce NO_3_‐N leaching by 6.1%.
- Reducing the maximum return to N value from 0.05 (N price/crop value ratio) to 0.1 and replacing 50% of corn‐corn with alfalfa‐corn in Bonanza Valley can reduce N fertilizer input by 27.9%.

## Plain Language Summary

Farmers in the Bonanza Valley of Central Minnesota, who irrigate their sandy fields, are losing a lot of nitrate nitrogen, which pollutes groundwater. To find solutions, the Environmental Policy Integrated Climate computer model was used to estimate how much nitrate was lost over 8 years. Several farm rotations were evaluated, including continuous‐corn (C‐C), corn‐soybean (C‐Sb), and alfalfa‐corn (A‐C). Nitrate‐N leaching losses were predicted with conventional versus reduced rates of nitrogen (N) fertilizer, without and with rye cover crops that take up excess N, and with conventional versus improved automated irrigation scheduling. A final scenario considered nitrate‐N leaching when half of the C‐C fields were switched to A‐C. The highest losses (about 52 kg ha^−1^) occurred in the C‐C rotation using the conventional fertilizer rate. However, if all these alternative practices were adopted across the 13,375 ha of irrigated sandy soil in the Valley, the total nitrate pollution would drop by 27.4%.

**Abbreviations**
A‐C: alfalfa‐corn
C‐C: continuous‐corn
C‐Sb: corn‐soybean
EEFlux: Earth Engine Evapotranspiration Flux
EPIC: Environmental Policy Integrated Climate
ET_c_
: crop evapotranspiration
MRTN: maximum return to N value
NRMSE: normalized root‐mean‐square error
NSE: Nash‐Sutcliffe coefficient

## 1 INTRODUCTION

Most corn (*Zea mays* L.) in US corn belt is grown in a continuous‐corn (C‐C) or corn‐after‐soybean (*Glycine max* L.) (C‐Sb) rotation (Struffert et al., 2016), where high rates of nitrogen (N) fertilizer applied to corn have relatively low recovery efficiencies ranging from 35% to 60%. The low crop recovery of supplemental N results in nitrate leaching into groundwater, streams, and estuaries (Randall et al., 1997; van Es et al., 2019). The US Midwest contributes to hypoxic zones in places such as Lake Erie (Watson et al., 2016) and the Gulf of America (Rabalais & Turner, 2019). The magnitude of N losses mainly depends upon weather, irrigation and N management practices, N mineralization from soil organic matter, soil properties, and crop management (Randall et al., 1997; Singh et al., 2023; Tahir et al., 2025; van Es et al., 2019).

In the Bonanza Valley of central Minnesota, NO_3_‐N pollution of groundwater is an ongoing challenge. Excessive irrigation and high use of N fertilizer for corn in the coarse‐textured soils of Bonanza Valley elevate the risk of nitrate contamination in groundwater (Pennino et al., 2020; Struffert et al., 2016). Sandy soils with limited available water capacity can cause severe soil water deficits that can only be offset by frequent irrigation (Smith & Westenbroek, 2015). The US Geological Survey reported a mean annual potential for groundwater recharge in most of the sand and gravel aquifers in Bonanza Valley ranging between 150 and 250 mm. About 40% of groundwater wells (<9 m deep) in central MN exceed the drinking water standard, while groundwater NO_3_‐N contamination trends have remained level over the last decade (Kroening & Vaughan, 2019). In the more vulnerable Bonanza Valley aquifer, 73% of 421 groundwater samples from 2000 to 2012 had NO_3_‐N levels above 10 ppm, and an increasing trend was observed over time (MNDNR, 2016).

Optimizing fertilizer N rates for agronomic and environmental considerations is a viable strategy for reducing NO_3_‐N leaching to groundwater. However, selecting optimal N fertilizer rates is surprisingly challenging, due to variation in potentially mineralizable N, soil characteristics, crop residue types, crop N fixation, and other management practices (Christianson & Harmel, 2015). N leaching losses are generally higher for C‐Cas compared to either corn‐soybean (C‐Sb) or corn‐alfalfa (*Medicago sativa* L.) rotations in the Midwest (Randall et al., 1997; Struffert et al., 2016). Thus, alternate cropping systems involving N fixation can help to reduce degradation of groundwater quality. However, a significant amount of NO_3_‐N leaching occurs in Minnesota during the fallow fall/spring season, following the harvest of corn or soybean. A winter rye (*Secale cereale* L.) cover crop has shown great potential to reduce NO_3_‐N leaching without significant reduction in corn yields (Feyereisen et al., 2006; Tahir et al., 2025).

A majority of the agricultural land under cultivation in the Bonanza Valley (82.9% out of 35,391 ha) has acquired groundwater pumping permits for crop irrigation (MNDNR, 2016). Therefore, accurate and efficient irrigation scheduling has become a major goal in getting optimum crop yield while minimizing NO_3_‐N leaching losses. Irrigation scheduling in Minnesota is generally practiced using the checkbook method (Steele et al., 2010), which tends to over‐irrigate crops (Orfanou et al., 2019). Under such circumstances, improved irrigation management scheduling can limit water use to sustainable levels (Singh et al., 2023; Wriedt et al., 2009).

Small experimental areas and short‐term monitoring under highly variable climatic conditions can produce misleading crop production and environmental degradation results, while long‐term monitoring is cost‐prohibitive. The Environmental Policy Integrated Climate (EPIC) is a field‐scale model capable of simulating biophysical and biogeochemical processes on a daily time step for hundreds of years, including crop productivity and environmental assessment, under various climatic scenarios (Lychuk et al., 2021; Wang et al., 2012; Williams et al., 1989). The EPIC model can perform long‐term simulations of crop yield, soil water, balance and soil N balance simultaneously (Balkovic et al., 2013; Wang et al., 2012; Williams et al., 1989). EPIC model can also estimate the irrigation water requirements of different crops using irrigation auto‐trigger at different soil water stress levels (Singh et al., 2023; Wriedt et al., 2009).

Estimation of crop productivity and its associated NO_3_‐N leaching at a large scale has attracted surging interest among agronomists and ecologists. The upscaling of a crop model from field to regional scale requires linking spatial and temporal scale variations of soil, weather, and management data. The integration of satellite observations of crop evapotranspiration (ET_c_) into crop models has been proposed as a means of improving yield estimations, as ET_c_ is a good integrator of weather, soil hydrology, and stress factors influencing crop growth and yield (Khan et al., 2019). METRIC Earth Engine Evapotranspiration Flux (EEFlux), a fully automated evapotranspiration mapping tool that operates on the Google Earth Engine platform, has the potential to assess water use in farm fields as well as large regions. EEFlux automated implementation of METRIC has shown promise as a tool to estimate ET_c_. The EEFlux platform applies METRIC energy balance model to Landsat series imagery (30 m resolution) to obtain instantaneous ET_c_, while the daily ET_c_ during the cloud‐free days is obtained using reference ET (Kadam et al., 2021; Venancio et al., 2020). ET_c_ estimates from remote sensing images and simple crop growth‐transpiration algorithms can be an alternative to the use of standalone crop models for real‐world yield assessment (Kadam et al., 2021).

Quantifying the relative impact of fertilizer N input under different crop rotations, with and without rye, and modeling auto‐irrigation management on crop yield and nitrate‐N leaching losses have been seldom studied at a regional scale on a long‐term basis. Objectives of this study were (a) to assess impacts of alternative cropping systems, N rates, rye as cover crop, and irrigation scheduling management effects in central Minnesota using EPIC simulated crop yield and NO_3_‐N leaching losses and (b) to upscale field‐level EPIC model predictions to the regional scale (Bonanza Valley) using METRIC‐EEFlux estimated ET_c_ and corresponding crop yield estimates, irrigation water use data, and soil characteristics.

**Core Ideas**

- Environmental Policy Integrated Climate (EPIC) model successfully simulated crop yield and NO_3_‐N leaching under alternative management scenarios.
- METRIC‐Earth Engine Evapotranspiration Flux, crop evapotranspiration, and irrigation water estimates were used to upscale the EPIC model predictions from field‐scale to the entire Bonanza Valley.
- The maximum return to N value at 0.1 (N price/crop value ratio), cover crop, and EPIC auto‐trigger irrigation reduced NO_3_‐N leaching in Bonanza Valley by 21.3% from baseline conditions.
- Shifting 50% corn‐corn to alfalfa‐corn in Bonanza Valley can reduce NO_3_‐N leaching by 6.1%.
- Reducing the maximum return to N value from 0.05 (N price/crop value ratio) to 0.1 and replacing 50% of corn‐corn with alfalfa‐corn in Bonanza Valley can reduce N fertilizer input by 27.9%.

## 2 MATERIALS AND METHODS

### 2.1 Field sites, soil, and weather input data for EPIC modeling

**FIGURE 1:**
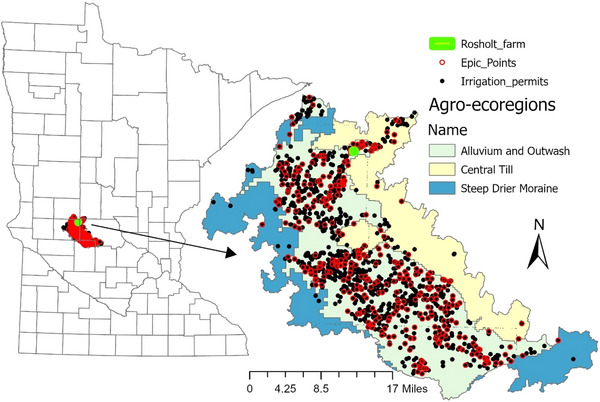
Distribution of agroecoregions, allocation of irrigation water permits, and Environmental Policy Integrated Climate (EPIC) model simulation fields in Bonanza Valley.

**FIGURE 2:**
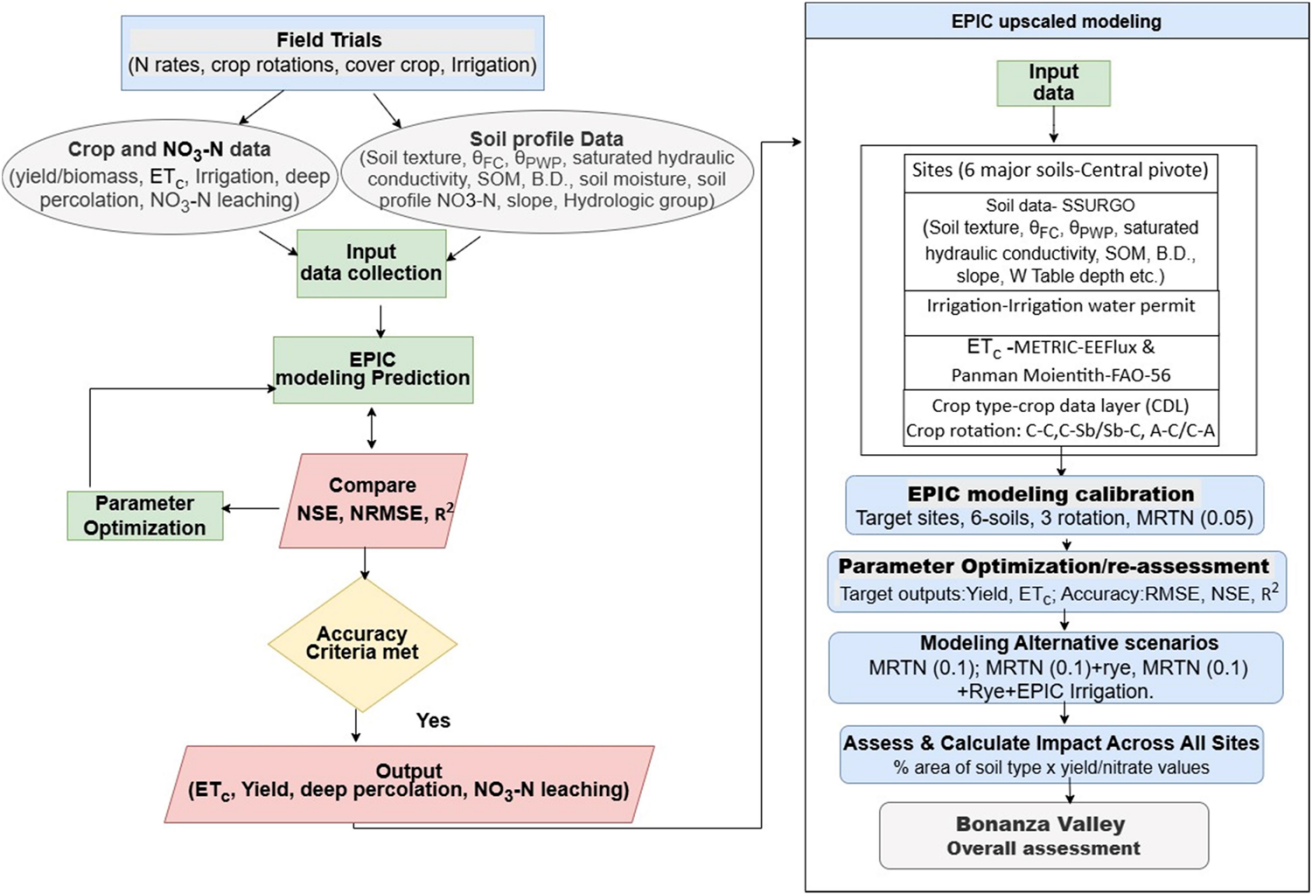
Schematic diagram showing Environmental Policy Integrated Climate (EPIC) modeling simulation and upscaling process for Bonanza Valley. A‐C, alfalfa‐corn; B.D., bulk density; C‐C, continuous‐corn; C‐Sb, corn‐soybean; ETc, crop evapotranspiration; MRTN, maximum return to N value; NSE, Nash‐Sutcliffe coefficient; SOM, soil organic matter; *θ*_FC_, volumetric water content at field capacity; *θ*_PWP_, volumetric water content at permanent wilting point.

EPIC model simulation of crop yield, soil water balance, and soil N balance was carried out for 8 years (2010–2017) of C‐C, C‐Sb/soybean‐corn, and alfalfa‐corn (A‐C)/corn‐alfalfa (C‐A) with and without rye as cover crop. Simulations were based on site‐specific model calibration with parameter optimization from research trial data. Details are provided in Supporting Information S2 (field trials data used for model calibration). Six dominant coarse‐textured soil series (Arvilla, Sandburg, Estherville‐Hawick, Estherville, Osakis, and Renshaw) under irrigation from Bonanza Valley with C‐C, C‐Sb/Sb‐C, and A‐C/C‐A crop rotations were selected for model simulation (Table 1). An area of 28,875 ha is irrigated in Bonanza Valley, among which 58.0% (16,762 ha) was under these three crop rotations of interest, and 46.3% (13,375 ha) was represented by the six soils targeted for EPIC simulation. A total of 322 center pivot irrigation fields were under these three crop rotations and six soils, from a total of about 969 water permits issued by the Minnesota Department of Natural Resources (DNR) in the Bonanza Valley (Figure 1). Soil physicochemical properties measured either at the start of the experiment or collected from the USDA SSURGO soils web survey (Table 1) are described in detail in the Supporting Information S3 (soil types, crop management, and irrigation scheduling). Daily weather data (Figure S1) were obtained from measurements at Rosholt Farm in Westport, Minnesota, or from the Midwestern Regional Climate Center records at Glenwood, Minnesota. ET_c_ of the plot‐scale experimental trials and different target sites was estimated using version 0.10.4 of EEFlux during the mid‐stage of the crop in the months of July–September, depending upon the availability of data on specific dates and cloudiness. The ET_c_ values thus obtained by this method were compared to field estimates of ET_c_ at experimental sites. Annual ET_c_ on daily basis at experimental sites was calculated using the Penman–Monteith FAO‐56 method. In general, ET_c_ values for at least four dates during each year were obtained and averaged for comparison. The difference in yield/biomass of target sites from those of experimental sites was then calculated using the relationship between relative yield reduction and relative ET_c_ reduction (Garg & Dadhich, 2014). The schematic diagram of the EPIC model simulation and upscaling process for Bonanza Valley is illustrated in Figure 2.

**TABLE 1:** Physicochemical properties of different soil types at different depths of Bonanza valley.

		Soil types					
Parameter	Depth (m)	Arvilla	Sandberg	E.‐Hawick	Estherville	Osakis	Renshaw
SOM (%)	0–0.35	1.88	1.50	1.66	2.89	2.87	1.77
	0.35–0.7	0.21	0.50	0.21	0.62	0.31	0.34
	0.70–1.2	0.20	0.21	0.20	0.40	0.2	0.25
B.D. (Mg m^−3^)	0–0.35	1.54	1.59	1.39	1.41	1.51	1.45
	0.35–0.7	1.63	1.65	1.49	1.47	1.64	1.49
	0.70–1.2	1.63	1.65	1.58	1.60	1.65	1.55
Texture (USDA)	0–0.35	SL	LS	SL	L	L	L
	0.35–0.7	S	S	LS	SL	LS	LS
	0.70–1.2	S	S	S	S	S	LS
*θ*_FC_ (m^3^m^−3^)^*^	0–0.35	0.21 (0.18)	0.14	0.18	0.30 (0.23)	0.27 (0.23)	0.27 (0.23)
	0.35–0.7	0.12 (0.10)	0.08	0.09	0.22 (0.16)	0.10	0.13
	0.70–1.2	0.07	0.08	0.07	0.07	0.07	0.07
*θ*_PWP_ (m^3^m^−3^)^*^	0–0.35	0.12 (0.09)	0.05	0.09	0.10	0.12	0.13 (0.10)
	0.35–0.7	0.05	0.03	0.04	0.08	0.04	0.05
	0.70–1.2	0.02	0.03	0.02	0.05	0.03	0.02
*K*_fs_ (mm h^−1^)	0–0.35	100.8	468	100.8	32.4	39.3	32.4
	0.35–0.7	590.8	741.5	291.6	154.6	806.2	305.6
	0.70–1.2	1015.0	1015.0	331.2	507.6	1015.2	331.2
CEC (Cmol_c_kg^−1^)	0–0.35	13.4	5.7	9.3	13.9	14.1	16.6
	0.35–0.7	5.9	2.3	3.4	9.9	5.0	3.7
	0.70–1.2	2.3	2.3	2.5	2.5	3.8	2.6
Hydrologic group	0.0–1.2	A	A	A	A	C	B
Slope (%)	–	0–2	2–6	2–6	0–2	0–2	1–6
W.T.D. (m)	–	>2	>2	>2	>1.8	>2	>2
Drainage class	0.0–1.2	S.E.D.	E.D.	S.E.D.	S.E.D.	M.W.D.	S.E.D.

Abbreviations: B.D., bulk density; CEC, cation exchange capacity; E.D., excessively drained; Es.‐Hawick, Estherville‐Hawick complex; *K*_fs_, field soil saturated hydraulic conductivity; L, loam; LCS, loamy coarse sand; LS, loamy sand; M.W.D., moderately well drained; S, sand; S.E.D., somewhat excessively drained; SL, sandy loam; SOM, soil organic matter; W.T.D., minimum water table depth; *θ*_FC_, volumetric water content at field capacity; *θ*_PWP_, volumetric water content at permanent wilting point.

* Values in parenthesis are calibrated during simulation.

### 2.2 N rates, alternative cropping systems, rye as cover crop, and EPIC auto‐irrigation scenarios

EPIC model simulation was carried out with five different alternative scenarios: (1) N applied (Table S1a) to corn at the maximum return to N value (MRTN) of 0.05 (N price/crop value ratio); (2) N applied to corn at reduced N fertilizer rates, that is, MRTN value of 0.1 (N price/crop value ratio); (3) adding rye cover crop at MRTN of 0.1; (4) using EPIC model auto‐trigger irrigating in scenario 3;, and (5) converting 50% of the C‐C acreage to A‐C in scenario 4. The MRTN values (0.05 and 0.1 N price/crop value ratio) were adopted from Kaiser et al. (2020), Kaiser et al. (2023), and Kaiser et al. (2025), which remained consistent with time. Crop and N management details are given in the Supporting Information S3 (soil types, crop management, and irrigation scheduling). Irrigation depth (Table 2) applied with a center pivot sprinkler system (at 90% efficiency) for each selected site was calculated based on the irrigation water permit usage (Figure 1; Figure S2) data and area (Figure S2) of that specific center pivot in the Bonanza Valley. For EPIC auto‐irrigation, locally calibrated (Table S2) EPIC model was shifted to auto‐irrigation option with a set of parameters given in Table S3. Matric potential values of EPIC irrigation auto‐trigger for loamy sand/sandy loam and loam soils (0–0.2 m) were set to −300, and −450 kPa, respectively. Total NO_3_‐N leaching load (t year^−1^) in Bonanza Valley under different crop rotations was calculated by multiplying NO_3_‐N leaching loss rate (kg ha^−1^ year^−1^) under a specific soil type and the area (ha) of that irrigated soil in Bonanza Valley.

**TABLE 2:** Irrigation water depth and soil water stress under different crops during the growing season for conventional irrigation management versus irrigation simulated using the Environmental Policy Integrated Climate (EPIC) auto‐trigger method.

	2010	2011	2012	2013	2014	2015	2016	2017	Mean
**(a)** **Irrigation** **(mm)** ^*^									
Corn	100.7	100.3	200.8	233.0	150.0	152.9	174.0	176.1	**161.0**
	(−17.4)	(5.9)	(−23.2)	(−41.2)	(2.3)	(−5.6)	(89.7)	(−70.3)	(−**7.5)**
Soybean	76.3	66.5	195.4	192.1	141.4	109.0	118.3	129.6	**128.6**
	(−14.5)	(12.3)	(−47.4)	(−38.7)	(−5.6)	(8.0)	(95.6)	(−43.4)	(−**2.2)**
Alfalfa	80.6	70.6	219.9	220.0	160.3	118.5	137.0	137.6	**143.1**
	(11.4)	(42.4)	(−19.6)	(−6.7)	(5.4)	(37.6)	(136.4)	(32.2)	**(31.1)**
**(b)** **Stress** **(d)** ^**^									
Corn	2.4	4.4	5.3	5.5	4.7	1.9	8.5	2.3	**4.4**
	(0.2)	(0.3)	(1.4)	(2.5)	(2.2)	(0.2)	(2.5)	(0.7)	**(1.4)**
Soybean	0.0	2.3	1.5	4.5	2.6	0.5	6.8	0.5	**2.3**
	(0.7)	(0.6)	(0.0)	(1.2)	(1.0)	(0.3)	(2.2)	(0.0)	**(0.7)**
Alfalfa	3.2	5.1	6.8	6.2	5.5	2.1	7.5	3.2	**5.5**
	(0.2)	(1.4)	(2.2)	(2.4)	(2.5)	(2.6)	(3.3)	(1.5)	**(3.0)**
**(c) Precipitation**	880.1	765.3	559.2	432.1	506.8	713.5	404.9	742.2	**625.5**

*Note*. Data are averaged over soil types, N rates, and rye/no rye treatment.

* Values in parentheses indicate the difference in irrigation (mm) under EPIC auto‐trigger from the conventional irrigation amount under water permits.

** Values in parentheses indicate the water stress days under EPIC‐auto trigger scenario.

### 2.3 Model calibration and validation, upscaling, and efficiency assessment

WinEPIC v8.0, the most recent EPIC model graphical interface, was used to assemble inputs and interpret outputs from a large number of target irrigated sites from the Alluvium and Outwash agroecoregion in Bonanza Valley. A few irrigated sites present in fine‐textured Central Till and Steep Drier Moraine agroecoregions were also included for assessment (Figure 1). Field experiments under different crop rotations were used for model calibration. The EPIC model was calibrated using a limited number of parameters, most sensitive to crop growth and yield as described by Wallach et al. (2014) and Tahir et al. (2025). Model parameter selection for parameter optimization of these crops (Supplemental Material S4. EPIC model calibration detail; Tables S2 and S3) was set based on prior calibration at similar sites of central Minnesota (Singh et al., 2023; Tahir et al., 2025) and literature data (Balkovic et al., 2013; Chung et al., 2001; Wang et al., 2005, 2012; Williams et al., 1989). The EPIC model was calibrated for C‐C and C‐Sb with and without rye as cover crop, using Rosholt experimental trial data (Supporting Information S2; Table S1b); while the EPIC alfalfa model was calibrated using above‐ground biomass (https://varietytrials.umn.edu/alfalfa) and soil properties (Table S1c) of Lamberton, Rosemount, and Becker sites. Further, EPIC model upscaled calibration for six soil types in the Bonanza Valley for C‐C, C‐Sb, and A‐C rotations was based on initial calibration of EPIC model for different crops at experimental sites (Supporting Information S2), followed by validation of target sites (Figure 1; Supporting Information S3) at MRTN of 0.05 (Table S1a), and on satellite‐estimated ET_c_ under different crop rotations with four replicate sites (Figure S3). The Landsat satellite estimated METRIC‐EEFlux ET_c_ values (http://eeflux‐level1.appspot.com) at target locations in Bonanza Valley, different from experimental field sites, were used for model upscaled calibration. ET_c_ was calculated using the EEFlux platform, which applies METRIC energy balance model to Landsat series imagery (30 m resolution) to obtain instantaneous ET_c_. Daily ET_c_ of the growing season is obtained using reference ET. During cloud‐free days, growing season images were selected to ensure accurate ET_c_ retrieval. EEFlux utilizes the automated internal calibration of ET_c_, a feature derived from METRIC, which identifies “hot” and “cold” reference pixels within each Landsat scene to calibrate sensible heat flux. The daily ET_c_ estimates were aggregated for each field polygon within the Bonanza Valley study area as key inputs to the crop yield modeling. Using weather, remote sensing estimated ET_c_, permitted irrigation water use (Figure S2), and SSURGO soil physicochemical properties, the locally calibrated EPIC model was upscaled to all 322 irrigated sites in Bonanza Valley (Figure 1) for the six selected soil types, under C‐C, C‐Sb, and A‐C rotations. EPIC was then run with an MRTN value of 0.1 and rye as cover crop for C‐C, C‐Sb, and A‐C crop rotations. In the next step, EPIC was run at these sites with auto‐trigger irrigation using an optimized parameter set (Table S3). The model outputs were used to assess crop yield, ET_c_, deep percolation losses, and NO_3_‐N leaching losses for 2010–2017. Data from the model simulation were extracted for each crop under different crop rotations. Evaluation of model efficiency was performed based on ET_c_/yield estimates, separately for the calibration and validation sites using Nash‐Sutcliffe coefficients (NSE values), normalized root‐mean‐square error (NRMSE), and coefficient of determination *R* ^2^), along with graphical comparisons of measured and predicted outputs during the calibration and validation process. Definitions for these equations (Loague & Green, 1991; Nash & Sutcliffe, 1970) are:

$$\mathrm{NSE}=1-\frac{\sum_{i=1}^{n}{\left(O_{i}-S_{i}\right)}^{2}}{\sum_{i=1}^{n}{\left(O_{i}-O_{m}\right)}^{2}}$$

$$\mathrm{NRMSE}=\frac{\mathrm{RMSE}}{O_{m}}\;\times \;100$$

where root mean square error (RMSE) is given by the following equation:

$$\mathrm{RMSE}=\sqrt{\frac{\sum_{i=1}^{n}{\left(O_{i}-S_{i}\right)}^{2}}{n}}$$

$$R^{2}=\frac{{\left[\sum_{i=1}^{n}\left(O_{i}-O_{m})(S_{i}-S_{m}\;\right)\right]}^{2}}{\sum_{i=1}^{n}{\left(O_{i}-O_{m})(S_{i}-S_{m}\;\right)}^{2}}$$

where *S_i_* is simulated data, *O_i_* is observed data, *n* represents the number of observations in the dataset, and *O_m_* and *S_m_* are means of observed and simulated data, respectively. NSE values above 0.75, 0.65, and 0.5 correspond to very good, good, and satisfactory model performance, respectively (Moriasi et al., 2007). Likewise, NRMSE values < 10, 10–20, and 20–30 indicate excellent, good, and fair results, respectively (Loague & Green, 1991).

### 2.4 Statistical analysis

Model outputs of crop yield/biomass, ET_c_, deep percolation, and NO_3_‐N leaching losses were subjected to statistical analysis to examine the effects of year, soil type, irrigation/nitrogen management, and crop rotation. The experimental design followed a complete factorial structure. The analysis was implemented using Python's statsmodels library, by using Python 3.12, with the following key packages: pandas, data manipulation and reshaping; statsmodels, analysis of variance implementation and statistical modeling; scipy, supporting statistical functions; and numpy, numerical computations. Effects were considered statistically significant at *α* = 0.05 level. Higher‐order interactions were interpreted to understand the complex relationships between agricultural management practices and environmental factors.

## 3 RESULTS AND DISCUSSION

### 3.1 EPIC model input parameter optimization, upscaling, and model accuracy

Crop growth parameter optimization (Table S2) was performed to address the effects of climate, N fertilizer management in corn, crops grown under different rotations, the addition of rye as a cover crop, and crops grown under different soil types. EPIC predictions of crop yield/biomass and soil water and N balances were very sensitive to crop parameters (Tahir et al., 2025), such as drought‐prone maximum leaf area (DMLA), harvest index, base temperature for plant growth, length of crop growth period before declining of leaf area, and N contents at 50% maturity (BN_2_) and full maturity (BN_3_). N contents in corn and soybean yield and biomass were optimized at slightly lower than the default values. Rye was harvested before maturity, and default values for N contents in rye (0.021) were calibrated to a slightly higher value (0.023), which itself was lower than field‐observed values (Ricks & Fernandez, 2019). These optimized crop parameters (Table S2) are consistent with a meta‐analysis of experimental results across the United States, indicating that crops on sandy soils have a lower response to applied N compared to fine‐textured soils (Tremblay et al., 2012).

The most influential soil parameters (Table 1) included water contents at permanent wilting (*θ*_WP_) and field capacity (*θ*_FC_), saturated hydraulic conductivity (*K*_fs_), and soil organic matter (SOM). Arvilla, Estherville, Osakis, and Renshaw soils' value for *θ*_WP_ and *θ*_FC_ at different depths were calibrated to be lower than the default values obtained from the SSURGO database (Table 1). Similarly, lower values of *θ*_WP_ and *θ*_FC_ at different depths were calibrated by Tahir et al. (2025), compared to the SSURGO database values. The SSURGO database is a reasonable starting source of soil properties for simulation of crop yield. Perez‐Quezada et al. (2003) successfully used the EPIC model to simulate within‐field variability in four crop rotations using SSURGO soil data. However, optimization of *θ*_WP_ and *θ*_FC_ was carried out as default values from SSURGO are produced using pedotransfer functions and may result in biased estimates of soil water balance (Wang et al., 2012). The PARM parameters in EPIC related to soil N mineralization, N volatilization, N fixation, surface runoff, and soil water were optimized to better fit the model under local conditions (Table S3). The details of parameter optimization are given in the Supporting Information S4 (EPIC model calibration detail; Table S2, EPIC calibrated values of plant growth and N uptake). The important EPIC parameters optimized to obtain accurate estimates for crop yield, water balance, and NO_3_‐N losses are generally in line with values used by Wang et al. (2011), Wang et al. (2012), and Tahir et al. (2025). We adopted a multi‐objective parameter optimization approach of the EPIC model to calibrate the yield/biomass, soil water balance, and soil N balance. This approach critically reduces uncertainty and improves ability to represent spatial and temporal variability (Tatsumi, 2016).

The EPIC model was used for auto‐irrigation of corn, soybean, and alfalfa crops in the Bonanza Valley, with parameter optimization (Table S3) related to auto‐irrigation frequency, amount, and deficit irrigation options. Soil water tension values of −300 and −450 kPa in the top 0.2 m soil were selected for EPIC auto‐irrigation of corn/soybean/alfalfa in loamy‐sand/sandy‐loam and loam soils, respectively. Irmak et al. (2001) and Irmak (2019) suggested a similarly low matric potential (−406 kPa for 0‐ to 30‐cm soil depth) for irrigation scheduling of corn to get maximum yield in loamy sand soil. EPIC model auto‐irrigation is based on the 20 cm upper soil layer in order to respond to surface soil layer drying. A majority of root zone water in the Bonanza Valley originates from rainfall (Table 2), allowing deficit irrigation to be applied to reduce deep percolation losses while maintaining crop yield.

Modeling assessments requires a comprehensive and transparent evaluation of their accuracy. EPIC model performance was evaluated using three different metrics, that is, NSE model efficiency, NRMSE, and *R* ^2^ values (Table 3). Results indicate that METRIC‐EEflux estimated ET_c_ was in close agreement with EPIC‐simulated ET_c_ for selected dates at calibration and validation sites (Figure S3). Therefore, METRIC‐EEFlux‐estimated ET_c_ was used to upscale the ET_c_‐based yield assessment at target sites for six dominant soils in the Bonanza Valley. Khan et al. (2019) found that crop models have significant limitations in estimating real‐world yields across regional scales; thus, METRIC evapotranspiration and simple growth algorithms can improve yield estimation of annual crops. Model efficiency was assessed based on the measured ET_c_/yield for the calibration and validation sites. The EPIC model was able to accurately simulate the ET_c_ and yield/biomass of corn, soybean, and alfalfa under different crop rotations (Table 3). EPIC predictions of the ET_c_/yield of corn, soybean, and alfalfa were excellent (NSE ≥0.70; NRMSE ≤15.6; and *R* ^2^ ≥0.83) during calibration and good (NSE ≥0.57; NRMSE ≤18.9; and *R* ^2^ ≥0.71) during validation. Model efficiency for rye crop ET_c_ or biomass for calibration and validation was good‐satisfactory (NSE ≥ 0.54; NRMSE ≤19.4; and *R* ^2^ ≥0.74) and acceptable (NSE≥0.41; NRMSE ≤23.2; and *R* ^2^ ≥0.55), respectively. Moriasi et al. (2007) suggested that NSE values above 0.75, 0.65, and 0.5 correspond to very good, good, and satisfactory model performance, respectively. Model efficiency during calibration was generally higher than that observed during validation. This is in line with Balkovic et al. (2013), who explained that better agreement can be achieved through the calibration of the model with limited parameter optimization, along with specific site information and management practices on crop.

**TABLE 3:** Evaluation of model accuracy for estimates of crop evapotranspiration and crop yield.

			Corn			Soybean	Alfalfa	Rye
			C‐C	C‐Sb	A‐C	C‐Sb	A‐C	C‐C/C‐Sb/A‐C
ET_c_	Calibration	NSE	0.91	0.79	0.75	0.87	0.76	0.76
		NRMSE	14.54	12.30	15.60	7.65	13.40	19.40
		*R* ^2^	0.87	0.84	0.92	0.89	0.90	0.78
	Validation	NSE	0.70	0.73	0.65	0.67	0.57	0.56
		NRMSE	15.50	16.50	18.90	12.34	7.99	23.40
		*R* ^2^	0.79	0.71	0.88	0.78	0.82	0.69
Yield	Calibration	NSE	0.87	0.76	0.87	0.95	0.70	0.54
		NRMSE	8.97	11.87	7.80	4.67	11.23	16.70
		*R* ^2^	0.89	0.83	0.94	0.96	0.84	0.74
	Validation	NSE	0.74	0.65	0.68	0.70	0.60	0.41
		NRMSE	13.40	9.99	14.30	11.50	15.40	23.20
		*R* ^2^	0.83	0.80	0.76	0.87	0.78	0.55

Abbreviations: A‐C, alfalfa‐corn; C‐C, continuous‐corn; C‐Sb, corn‐soybean; NRMSE, normalized root‐mean‐square error; NSE, Nash‐Sutcliffe coefficient.

### 3.2 Simulation results

Well‐calibrated and validated EPIC model predictions of crop yield, ET_c_, deep percolation, and NO_3_‐N leaching losses under the baseline management scenario involved long‐term (2010–2017) simulations at MRTN of 0.05 rates. These baseline results were compared with results from alternative fertilizer N inputs (MRTN of 0.1), rye management, EPIC scheduling of irrigation using auto‐trigger, and alternative crop rotation scenarios. Results in the sections below are presented as simulated outputs of the up‐scaled model from all 322 irrigated sites (13,375 ha) in Bonanza Valley under C‐C, C‐Sb, and A‐C crop rotations.

#### 3.2.1 Soil water input and soil water stress under conventional irrigation and EPIC irrigation scheduling

Precipitation and irrigation varied greatly across years (Table 2; Figure S1). Precipitation over an 8‐year simulation period averaged 625.5 ± 173.3 mm, ranging from a minimum value of 404.9 mm to a maximum value of 880.1 mm observed during the years 2016 and 2010, respectively. Soil water input under conventional irrigation averaged 161.0 ± 45.8, 128.6 ± 47.4, and 143.1 ± 56.0 mm for corn, soybean, and alfalfa, respectively, with maximum values of 233.0, 192.1, and 220.0 mm, applied during the dry year of 2013. EPIC model auto‐trigger reduced annual irrigation water input by 7.5 and 2.2 mm for corn and soybean, respectively; however, an increase of 31.1 mm was observed for the alfalfa. Meanwhile, model simulations indicate that soil water stress for all crops was reduced with EPIC auto‐irrigation scheduling due to better irrigation management compared to conventional irrigation. EPIC auto‐trigger water input with alfalfa was 20.9% higher than with conventional irrigation, implying that alfalfa was under‐irrigated. Current irrigation scheduling methods should be reassessed to increase alfalfa forage yield. Irrigation needs in Minnesota are typically estimated using the checkbook method. Previous studies indicated that the checkbook method for corn/soybean usually over‐applies irrigation in wet years, while it causes soil water stress by under‐irrigating crops during dry years (Orfanou et al., 2019). EPIC auto‐trigger irrigation used less water than the checkbook method for row crops and thus successfully provided an effective irrigation management method to alleviate soil desiccation. EPIC has previously shown promise to reliably estimate the crop yield, ET_c_, and soil water content (Guo et al., 2021; Singh et al., 2023; Wriedt et al., 2009).

#### 3.2.2 Crop yield and biomass

**FIGURE 3:**
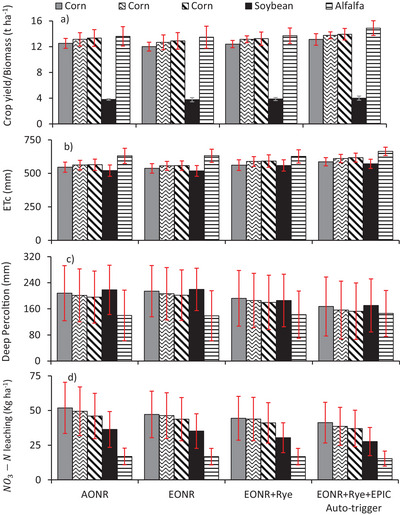
Crop yield or biomass, evapotranspiration rate, deep percolation, and nitrate leaching losses under different crop rotations and management scenarios. Note. Red bars indicate the standard deviation of six soil types with four replications for the years 2010–2017. A‐C, alfalfa‐corn; C‐C, continuous‐corn; C‐Sb, corn‐soybean; EPIC, Environmental Policy Integrated Climate.

The yield of corn and soybean, forage biomass of alfalfa (Figure 3a; Table 4; Figure S4), and rye biomass (Table S4) were significantly affected by alternative N, crop rotation, and irrigation management practices, soil types, and year. Corn yield at MRTN of 0.05 averaged 5.0 and 6.6% higher under C‐Sb and A‐C rotations, respectively, compared to yields with C‐C (12.5 t ha^−1^). Applying MRTN of 0.1 decreased the corn yield by 4.1%, 3.5%, and 3.3% under C‐C, C‐Sb, and A‐C rotations, respectively, without affecting the succeeding soybean yield (3.8 t ha^−1^). Long‐term establishment of rye and its in‐field termination increased the yield of corn and soybean by 3.2% and 2.7%, respectively. Increased corn and soybean yield with rye was a result of improvements in soil properties, including higher soil water storage, increased SOM, and lower NO_3_‐N leaching with cover crops (Basche et al., 2016; Tahir et al., 2025). Everett et al. (2019) conducted cover crop experiments at 19 sites in the Upper Midwest, indicating that subsequent release of N from rye residue is partially responsible for the lack of yield reduction of corn. Likewise, no reduction in soybean yield was observed following a rye cover crop at the Rosemount and Waseca locations in Minnesota (De Bruin et al., 2005). Rye produced biomass values of 0.89, 0.99, 1.2, and 2.1 t ha^−1^, and N uptake of 20.3, 22.7, 28.1, and 47.8 kg ha^−1^ (Table S4), when planted after corn (C‐C), corn (C‐Sb), corn (A‐C), and soybean (C‐Sb), respectively. Rye planted after soybean resulted in higher biomass and N uptake than rye planted after corn. Higher soil residual N available for a succeeding rye cover crop due to biological N fixation and N release (accompanied by less immobilization) from soybean residue caused higher rye biomass and N uptake (Basche et al., 2016). Moreover, rye after soybean shows a higher plant population due to less shading compared to corn (Tahir et al., 2025). Spatio‐temporal variations in crop yield and rye biomass were observed due to variations in soil properties and extreme weather conditions, thereby influencing the NO_3_‐N and soil available water. Crop yield/biomass (Table 4; Figure S4) was lower in the coarser textured soils and increased in the following order: Sandberg < Estherville‐Hawick complex ≈ Arvilla ≤ Renshaw ≈ Estherville ≤ Osakis. Coarser‐texture soils had lower SOM, lower soil water retention, and were excessively drained (Table 1), limiting their fertility, increasing vulnerability to drought, and NO_3_‐N leaching losses, compared to the medium‐textured soils. Irrigation management with EPIC auto‐trigger lowered crop water stress and increased corn yield by 5.2%, soybean yield by 3.5%, and alfalfa forage biomass by 8.6%, compared to conventional irrigation. Results indicated that using better irrigation management and incorporation of rye can offset the effect of reduced N rates on crop yield/biomass. Meanwhile, reduced N rates (MRTN of 0.1) minimize environmental concerns of nitrate leaching, while the incorporation of rye biomass also improves soil health. Rubin et al. (2016) found a negligible impact of MRTN at 0.05 in increasing N availability for sandy soils of Minnesota and estimated corn MRTN at 0.1 of 233 kg ha^−1^ under C‐C, while MRTN at 0.1 for corn was 49 kg ha^−1^ lower in a C‐Sb rotation. Our simulation results also indicate that reducing N rates of corn from MRTN at 0.05 to MRTN at 0.1 under A‐C and C‐Sb caused a small <4.0% yield reduction. MRTN at 0.1 in the C‐C rotation had a higher corn yield reduction of 5.5%; however, C‐C causes high nitrate leaching, and thus it is not recommended in sandy soils of Central Minnesota (Struffert et al., 2016; Tahir et al., 2025). Corn MRTN at 0.1 under C‐Sb/A‐C was not only much lower than with C‐C, reducing fertilizer input costs, but also produced a higher corn yield. Fertilization of corn at different N rates did not affect the succeeding soybean yield. Earlier findings also indicate that soil‐available N did not affect soybean yield due to the zero requirement for soybean N fertilizer (Schmitt et al., 2001). Corn in an A‐C rotation receiving reduced N application rates of 80 kg ha^−1^ in the first year had a yield higher than in the C‐C rotation. Yost et al. (2012) indicated a similar MRTN at 0.1 value of 85 kg ha^−1^ for the first‐year corn following alfalfa, with an N reduction of 168 kg ha^−1^, compared to continuous corn. For the second‐year corn after alfalfa, N fertilizer input was reduced by 80 and 85 kg ha^−1^ under MRTN at 0.05 and 0.1, respectively, compared to corn under C‐C rotation. A similar credit of 150 and 75 kg N ha^−1^ from alfalfa was recommended for the first and second years of corn in Minnesota (Coulter et al., 2012). Results imply that A‐C rotation should be adopted in the Central Sands of Minnesota to reduce fertilizer N input costs and nitrate leaching losses (Tahir et al., 2025). A higher yield of corn and soybean, as well as forage‐biomass of alfalfa observed under soil‐type‐based EPIC auto‐trigger scheduling, was a result of better synchronization between crop demand and irrigation water availability (Table 2). Previously, a well‐calibrated EPIC model has proved to be very good at simulating the irrigation demand and resulting yield of different row crops (Choruma et al., 2021; Guerra et al., 2004, 2005; Ko et al., 2009; Singh et al., 2023).

**TABLE 4:** Results (*p* values) of statistical analysis of crop yield, crop evapotranspiration (ET_c_), deep percolation, and nitrate‐N leaching losses response to year, soil type, N and irrigation management, and crop rotation.

Source of variation	Yield/biomass	ET_c_	Deep percolation	Nitrate leaching
Year (Y)	<0.001	<0.001	<0.001	<0.001
Soil type (S)	<0.01	<0.01	<0.001	<0.001
Irrigation and N management (NI)	<0.01	<0.001	<0.001	<0.01
Crop rotation (CR)	<0.001	<0.01	<0.01	<0.001
**Interactions**				
Y × S	<0.001	0.8577	<0.001	<0.001
Y × NI	≥1.00	<0.001	<0.001	<0.01
Y × CR	<0.001	<0.001	<0.001	<0.001
S × NI	0.759	<0.001	<0.01	<0.01
S × CR	<0.01	0.912	<0.001	<0.001
NI × CR	<0.001	<0.001	<0.001	<0.01
Y × S × NI	≥1.000	≥1.000	≥1.000	≥1.000
Y × S × CR	<0.001	≥1.000	≥1.000	≥1.000
S × NI × CR	0.998	≥1.000	<0.001	≥1.000
Y × S × NI × CR	≥1.000	≥1.000	≥1.000	≥1.000

*Note*: *p*‐value ranges of 0.000–0.001, 0.001–0.01, 0.01–0.05, and < 0.05 indicate extremely significant, very significant, significant, and non‐significant results, respectively.

#### 3.2.3 Crop evapotranspiration rates

ET_c_ was affected by N rates, crop types, cover crop, soil type, year, and irrigation management practices (Figure 3b; Table 4; Figure S4). C‐C under MRTN of 0.05 averaged 546.3 mm annual ET_c_, while an increase of 2.8 and 3.3% was observed when corn was planted under C‐Sb and A‐C rotation, respectively. These increases in ET_c_ were attributed to the higher yield of corn when grown in rotation with soybean and alfalfa, compared to C‐C (Figure 3a). Soybean (C‐Sb) and alfalfa (A‐C) with MRTN of 0.05 had average annual ET_c_ values of 520.7 and 630.5 mm, respectively. Corn at MRTN of 0.1 showed slightly lower ET_c_ (7 mm), compared to MRTN of 0.05. Planting rye after corn in C‐C, C‐Sb, and A‐C rotation increased ET_c_ by 4.5%, 5.8%, and 6.1%, respectively, while a boost of 7.8% was observed when rye was planted after the soybean crop. Higher ET_c_ observed with rye can help to reduce deep percolation losses and NO_3_‐N leaching during the fallow period. Compared to conventional irrigation, irrigation scheduling using EPIC auto‐trigger increased annual ET_c_ by 4.2%, 2.3%, and 6.0% under corn, soybean, and alfalfa, respectively. Garcia y Garcia and Strock (2018) also observed a similar pattern of ET_c_ with slightly lower values for corn (535 mm), soybean (484 mm), and alfalfa (625 mm), with a lower water input (690 mm) at Lamberton, Minnesota. EPIC model can be used to accurately assess ET_c_ and thus the impact on crop yield during droughts associated with a changing climate (Guo et al., 2021). Thus, proper irrigation can lessen the negative effects of a warming climate on corn production (Irmak et al., 2022). Quicker accumulation of growing degree days resulting from global warming tends to increase water deficits during reproductive (R1–R6) stages (Prasad et al., 2018). These changes can be addressed with EPIC auto‐irrigation to better match irrigation amounts with plant water requirements. Higher alfalfa crop ET_c_ under EPIC irrigation resulted in higher water input and lower soil water stress (Table 2), compared to conventional irrigation, where alfalfa was apparently under‐irrigated. Soil type also had a significant effect on ET_c_ (Table 4; Figure S4). Annual ET_c_ increased in the following order: Sandberg < Arvilla ≤ Estherville‐Hawick complex ≤ Renshaw ≤ Estherville < Osakis. Soils with loam soil surface texture showed higher ET_c_ compared to loamy sand/sandy loam soil. Coarser‐textured soils had lower soil water retention (Table 1) and resulted in lower ET_c_, thereby producing lower crop yield/biomass.

#### 3.2.4 Deep percolation

Study sites were irrigated by sprinkler irrigation based on the checkbook method of estimating ET_c_ demand. Heavy rainfall events caused high deep percolation losses in highly permeable, low water‐retaining sandy soils. High variability of deep percolation was observed across years, as indicated by standard deviation values in Figure 3c, implying that these losses were primarily driven by heavy rainfall events. Deep percolation losses were, however, reduced by alternative management practices such as a perennial alfalfa crop rotation, rye cover crop, and better irrigation management with EPIC. Soil type also had a significant effect on deep percolation losses (Table 4; Figure S4). Deep percolation losses decreased in the following order: Sandberg > Arvilla ≥ Estherville‐Hawick complex ≥ Estherville ≥ Renshaw > Osakis. Excessively drained coarser‐textured soil showed higher deep percolation losses compared to the medium‐textured moderately drained soils. Deep percolation losses under different crops decreased in the following sequence: soybean > corn > alfalfa. C‐C under conventional irrigation at MRTN of 0.05 averaged 208.0 mm deep percolation losses; however, when corn was planted after soybean and alfalfa, the amount was reduced by 3.2% and 5.8%, respectively. This reduction was caused by higher ET_c_ rates when corn was planted after soybean or alfalfa, compared to continuous corn. Soybean and alfalfa under conventional irrigation at MRTN of 0.05 showed the highest (218.2 mm) and lowest (139.8 mm) deep percolation losses in response to the lowest and highest ET_c_ observed under these crops (Figure 3b). Slightly increased deep percolation losses were observed in corn under MRTN of 0.1; however, planting rye cover crop reduced these losses by 10.4% and 15.5% under corn and soybean, respectively. A notable increase in ET_c_ is observed with rye during fall and early spring, thereby reducing deep percolation losses (Tahir et al., 2025). Rye planted after soybean was more efficient in reducing deep percolation losses due to higher biomass production, compared to rye planted after corn. EPIC irrigation scheduling management decreased deep percolation losses by 14.7% and 8.1% under corn and soybean, respectively. EPIC irrigation scheduling for alfalfa did not cause any noticeable decrease in deep percolation losses; however, higher water input caused a boost in alfalfa forage biomass, compared to the checkbook method. Previous studies also found that annual deep percolation is generally high in the sandy soil of Minnesota, with an annual groundwater recharge rate of 150–254 mm in the Bonanza Valley sands and gravel aquifer (Smith & Westenbroek, 2015). In Rosemount, Minnesota, slightly lower deep percolation values ranging from 145 to 202 mm year^−1^ were observed under C‐C or C‐Sb rotations in fine silt overlaying a sandy soil for a 30‐year period with an average annual water input of 887 mm (Ochsner et al., 2017). Soil at our study site was coarser in texture; however, it received lower annual water inputs, ranging from 751.1 to 786.5 mm and 746.9 to 803.0 mm under checkbook and EPIC irrigation scheduling, respectively, for a range of alternative crops.

#### 3.2.5 Annual nitrate‐N leaching losses and overall assessment

**FIGURE 4:**
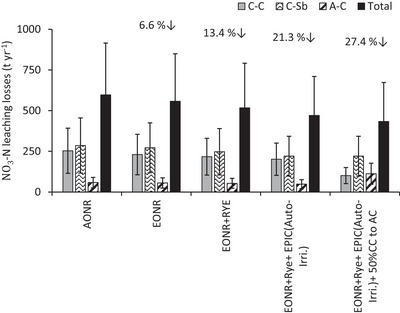
Comparison between annual NO_3_‐N loadings to groundwater for baseline versus alternative management practices in the irrigated area of the Bonanza Valley region in Minnesota. Downward percent changes in total nitrate‐N leaching for each scenario are provided relative to the baseline maximum return to N value (MRTN) of 0.05 scenario. Note. Standard deviation bars represent the annual variation. A‐C, alfalfa‐corn; C‐C, continuous‐corn; C‐Sb, corn‐soybean; EPIC, Environmental Policy Integrated Climate.

Leaching losses of NO_3_‐N were significantly affected by crop rotation, N and irrigation management, soil type, and different years of the study period (Table 4; Figure 3d; Figure S4). Annual NO_3_‐N leaching losses under corn with conventional irrigation and MRTN of 0.05 averaged 51.9, 49.5, and 46.1 kg ha^−1^, when planted in C‐C, C‐Sb, and A‐C rotations, respectively. Comparatively lower NO_3_‐N leaching losses of 36.4 and 16.9 kg ha^−1^ were observed under soybean and alfalfa when planted under C‐Sb and A‐C rotations, respectively. MRTN rate of 0.1 decreased the NO_3_‐N leaching losses by 9.1%, 6.3%, and 4.9% when corn was planted in C‐C, C‐Sb, and A‐C rotations, respectively. At MRTN of 0.1, planting rye after corn and soybean reduced NO_3_‐N leaching by 5.8% and 13.6%, respectively. EPIC auto‐irrigation at MRTN of 0.1 caused further decreases of 9.6%, 9.1%, and 8.5% in NO_3_‐N leaching losses for these crops. Overall, NO_3_‐N leaching losses of 597.3 t year^−1^ (Figure 4) occurred in the 13,375 ha irrigated area of Bonanza Valley (Figure S5) under C‐C/C‐Sb/A‐C rotation at MRTN of 0.05. These leaching losses were reduced by 6.6% at MRTN of 0.1. Adding rye after corn and soybean at MRTN of 0.1 further reduced leaching losses by 13.4%, while shifting conventional irrigation scheduling to EPIC auto‐irrigation reduced these losses by 21.3%. A total reduction of 27.4% in NO_3_‐N leaching losses was achieved when 50% of C‐C acres were converted to A‐C with EPIC auto‐irrigation, MRTN of 0.1, and a rye cover crop. Considering the environmental cost of 18.54 US$ kg^−1^ NO_3_‐N losses for growing crops (Singh et al., 2025), these alternative scenarios collectively can reduce the annual financial burden by $3.04 million in Bonanza Valley. Moreover, fertilizer N input of 2204 t year^−1^ in the 13,375 ha of irrigation in Bonanza Valley can be reduced by 12.7% and 27.9% when MRTN of 0.05 is replaced by MRTN at 0.1% and 50% C‐C is replaced by A‐C at the MRTN of 0.1 rate, respectively (Figure S5). Considering the average N price during 2010–2017 (Bruening, 2025), these reduced N inputs in Bonanza Valley can save farmers an additional $690,000 annually. Soil type also had a significant effect on NO_3_‐N losses (Table 4; Figure S4). NO_3_‐N losses decreased in the following order: Sandberg > Arvilla ≥ Estherville‐Hawick complex ≥ Estherville ≥ Renshaw > Osakis. Bonanza Valley receives major soil water input in the form of precipitation, leaching to high deep percolation losses (Figure 3). Under these conditions, excessively drained coarser texture soil showed higher NO_3_‐N leaching losses, compared to the medium‐textured, moderately drained soils.

High NO_3_‐N leaching losses predicted in this study under conventional irrigated corn at MRTN of 0.05 confirm the widespread finding of high NO_3_‐N concentrations in groundwater over the last two decades in the sandy irrigated areas of central Minnesota (Kroening & Vaughan, 2019). Large reductions in NO_3_‐N leaching losses with an alternative A‐C rotation also confirm previous findings that alfalfa significantly reduces NO_3_‐N concentrations in the soil profile (Randall et al., 1997). Rye also proved very effective in reducing NO_3_‐N leaching losses, especially when planted after a soybean crop. At Westport, Minnesota, similar reductions of 6.0% and 15.6% were observed when rye was planted after corn and soybean for a decade, respectively (Tahir et al., 2025). Residual soil NO_3_‐N in the top 60 cm soil suggests a risk for nitrate leaching during the non‐growing season (Bohman et al., 2020). Rye cover crop takes up soil N in biomass during the late fall and early spring, thereby reducing NO_3_‐N leaching losses (Tahir et al., 2025). Rye thus can conserve soil N by altering N dynamics, ultimately increasing the crop‐available N (Everett et al., 2019; Pantoja et al., 2016).

The EPIC model successfully estimated the irrigation water requirement of different crops. Therefore, EPIC auto‐irrigate at different soil water stress levels can reduce deep percolation losses (Wriedt et al., 2009). EPIC auto‐irrigation scheduling of corn at Becker and Westport sites of central Minnesota also indicated much lower irrigation amount and resulting deep percolation losses at ‐300 kPa and ‐450 kPa matric potential auto‐trigger in sandy loam and loamy sand soil, respectively, compared to checkbook/soil moisture‐based irrigation (Singh et al., 2023). High variation in NO_3_‐N leaching was observed across, years as indicated by large standard deviations (Figure 3d). The EPIC model was good in coping with effects of annual variations in precipitation and irrigation water input on crop yield, deep percolation, and NO_3_‐N leaching losses. Results imply that EPIC modeling of yield versus NO_3_‐N leaching losses in response to water and N fertilizer management under alternative crop rotation and rye can play an increasingly important role in long‐term strategies for minimizing the effects of agriculture on water quality while maintaining the optimum crop yield.

## 4 CONCLUSIONS

Achieving nitrate‐N leaching reduction targets requires accurate long‐term and regional‐scale assessment of crop yield versus NO_3_‐N leaching losses in response to different management scenarios related to crop rotation, cover crop, fertilizer N, and irrigation. The EPIC model was accurately calibrated for C‐C, C‐Sb, and A‐C rotations, receiving an MRTN of 0.05 and an MRTN of 0.1; addition of rye cover crop at an MRTN of 0.1; and EPIC auto‐trigger irrigation at an MRTN of 0.1 with rye. The calibrated model was successfully upscaled to Bonanza Valley using METRIC EEFlux ET_c_ estimates, irrigation water, and soil data. Results indicate that C‐Sb and A‐C rotation at MRTN of 0.05 reduced NO_3_‐N leaching by 17.3% and 39.4%, respectively, compared to C‐C rotation (51.9 kg ha^−1^). Reducing corn N fertilization rates to MRTN at 0.1 under different rotations decreased NO_3_‐N leaching under corn by 6.8%, with only a 3.6% reduction in yield. Rye reduced NO_3_‐N leaching losses quite effectively (5.8%) after corn and impressively after soybean (13.6%), while showing a slight improvement in crop yield. EPIC auto‐trigger irrigation not only improved crop yield but also decreased NO_3_‐N leaching rates by 9.6%, 9.1%, and 8.5% under corn, soybean, and alfalfa, respectively. In the Bonanza Valley, an overall reduction of 27.4% in NO_3_‐N leaching can be achieved without significantly reducing crop yield through using MRTN of 0.1 for MRTN of 0.05, planting cover crop, replacing conventional irrigation with EPIC auto‐trigger, and replacing 50% of C‐C with A‐C rotation. Results also imply that once calibrated and up‐scaled for different crops and soil types, the EPIC model is useful as a tool for regional planning of N and irrigation management, cover crop management, and adopting alternative crop rotations to protect groundwater quality.

## AUTHOR CONTRIBUTIONS

**Muhammad Tahir**: Conceptualization; data curation; formal analysis; methodology; writing—original draft; writing—review and editing. **David J. Mulla**: Conceptualization; funding acquisition; investigation; methodology; project administration; resources; supervision; validation; visualization; writing—review and editing.

## CONFLICT OF INTEREST STATEMENT

The authors declare no conflicts of interest.

## Supporting information



Tables and detailed description of fertilizer N input under different crop rotations, historical crop rotation information, and cultural practices. Table and description of soil physicochemical properties of alfalfa experimental trial sites used for model calibration. Detailed description of Bonanza Valley soil, crop, and irrigation management. Tables and detailed description of EPIC model calibrated values of plant growth, N uptake, PARM file, and EPIC auto‐irrigation. Table of rye biomass and N uptake. Figures related to climate data. Figure of spatial distribution of crop rotation, irrigation depth, and area under each central pivot system in the Bonanza Valley. Figure showing METRIC‐EEFlux measured (M) vs EPIC simulated (S) ET_c_ values. Figure showing crop yield/biomass, ET_c_, deep percolation, and nitrate‐N leaching losses under different soil types. Figure showing crop area and fertilizer N input under different crop rotations in Bonanza Valley.
