# Supplementary material for: Linking remote sensing with crop modeling for yield and nitrate leaching predictions in Minnesota
Source: J Environ Qual. 2026 Jan 10;55(1):e70137. doi: 10.1002/jeq2.70137 (PMC12789989; doi:10.1002/jeq2.70137)
Supplement: Supplementary file 1 — Tables and detailed description of fertilizer N input under different crop rotations, historical crop rotation information, and cultural practices. Table and description of soil physicochemical properties of alfalfa experimental trial sites used for model calibration. Detailed description of Bonanza Valley soil, crop, and irrigation management. Tables and detailed description of EPIC model calibrated values of plant growth, N uptake, PARM file, and EPIC auto‐irrigation. Table of rye biomass and N uptake. Figures related to climate data. Figure of spatial distribution of crop rotation, irrigation depth, and area under each central pivot system in the Bonanza Valley. Figure showing METRIC‐EEFlux measured (M) vs EPIC simulated (S) ETc values. Figure showing crop yield/biomass, ETc, deep percolation, and nitrate‐N leaching losses under different soil types. Figure showing crop area and fertilizer N input under different crop rotations in Bonanza Valley. [file JEQ2-55-0-s001.docx]

Supplementary Material

**TABLE S1a** Fertilizer nitrogen inputs to corn, soybean, and alfalfa in the Bonanza Valley for eight years.

| Crop | --------------Corn----------------- | | | Soybean | Alfalfa |
| --- | --- | --- | --- | --- | --- |
| N rates | C-C | C-Sb | A-C **^*^** | C-Sb | A-C **^*^** |
| MRTN of 0.05 (N price/crop value ratio) | 260 | 235 | 80,180,260,260 | 0 | 35,0,0,0 |
| MRTN of 0.1 (N price/crop value ratio) | 225 | 200 | 80,140,225,225 | 0 | 35,0,0,0 |

MRTN, the maximum return to N value **^*^**Four values are for first to fourth year; Note. Recommendations based on UMN Ext Guidelines according to Kaiser et al. (2020), Kaiser et al. (2023), and Kaiser et al. (2025).

S1. Fertilizer N rates under different crop rotations

For continuous corn, 260 and 225 kg N ha^-1^ were applied under MRTN of 0.05 and MRTN of 0.1 (Table, S1a; Kaiser et al., 2020), while rates 25 kg ha^-1^ lower were considered for corn under C-Sb rotation. No fertilizer was applied to soybean, while alfalfa received 35 kg N ha^-1^ in the first year of an extended rotation. N rates applied to corn following termination of the extended alfalfa rotation were 80 kg ha^-1^, with rates in the second year following alfalfa of 140 and 180 kg ha^-1^ at MRTN of 0.1 and MRTN of 0.05, respectively (Table S1a). Corn received N in three to four splits.

**TABLE S1b** Field trials and N rates at Westport, MN used for EPIC model calibration for corn/soybean/rye sown under different crop rotations

|  |  | Fertilizer N (corn) | | ------------Crop rotation----------- | | |
| --- | --- | --- | --- | --- | --- | --- |
| Experiment type | Year | Calibration | Validation |  |  |  |
| N rates* | 2011-2015 | 270 | 225 | C-C | Sb-C | C-Sb |
| Cover crop** | 2015-16 & 2016-17 | 250 | 200 | Rye/no rye-C-C | Rye/ no rye-Sb-C | Rye/no rye-C-Sb |
| Irrigation*** | 2019-2021 | 270 | 270 | Auto-Irri/  CB | Auto-Irri/  CB | Auto-Irri/  CB |

*Experimental treatment used for EPIC calibration/validation from Rubin et al. (2016); During the year 2015, however, corn in both rotations was grown with recommended N (220 kg N ha^-1^) rates; **Experimental treatment used for EPIC calibration of rye from Ricks and Fernandez (2019) with N rates of 250 and 200 kg N ha^-1^ used for calibration and validation respectively, and; ***Experimental treatment used for EPIC calibration and validation of irrigation at farmer’s practices (Checkbook method), and EPIC-auto-irrigation, respectively, adopted from Singh et al. (2023); Note. Soil at the experimental site was Arvilla sandy loam.

**TABLE S1c** Soil physico-chemcial properties at alfalfa experimental sites used for model calibration

| Location | Depth | Lamberton | Rosemount | Becker |  |
| --- | --- | --- | --- | --- | --- |
| Soil Type | (m) | Normania^1^ | Waukegan^2^ | Hubbard-Mosford^3^ |  |
| Soil organic matter (%) | 0-0.35 | 2.63 | 3.33 | 1.50 |  |
|  | 0.35-0.7 | 1.00 | 0.50 | 0.87 |  |
|  | 0.70-1.2 | 0.25 | 0.19 | 0.23 |  |
| Bulk density (Mg m^-3^) | 0-0.35 | 1.30 | 1.45 | 1.55 |  |
|  | 0.35-0.7 | 1.34 | 1.45 | 1.59 |  |
|  | 0.70-1.2 | 1.45 | 1.60 | 1.60 |  |
| Texture (USDA) | 0-0.35 | Loam | Silt loam | Loamy sand |  |
|  | 0.35-0.7 | Loam | Silt loam | Loamy sand |  |
|  | 0.70-1.2 | Loam | Sand | Sand |  |
| θ_FC_ (m^3^m^-3^) | 0-0.35 | 0.30(0.27) | 0.31(0.26) | 0.16(0.15) |  |
|  | 0.35-0.7 | 0.28(0.24) | 0.29(0.22) | 0.15(0.10) |  |
|  | 0.70-1.2 | 0.28(0.18) | 0.09(0.09) | 0.14(0.08) |  |
| θ_PWP_ (m^3^m^-3^) | 0-0.35 | 0.16(0.13) | 0.17(0.14) | 0.06 |  |
|  | 0.35-0.7 | 0.14(0.11) | 0.15(0.12) | 0.05 |  |
|  | 0.70-1.2 | 0.13(0.09) | 0.04(0.05) | 0.02 |  |
| K_fs_ (mm hr^-1^) | 0-0.35 | 33.0 | 32.4 | 324.0 |  |
|  | 0.35-0.7 | 33.0 | 32.4 | 324.0 |  |
|  | 0.70-1.2 | 33.0 | 325.2 | 324.0 |  |
| CEC (Cmol_c_kg^-1^) | 0-0.35 | 21.5 | 18.3 | 6.5 |  |
|  | 0.35-0.7 | 19.6 | 14.5 | 5.6 |  |
|  | 0.70-1.2 | 15.8 | 3.7 | 1.7 |  |
| Hydrologic Group | 0.0-1.2 | C | B | A |  |
| Slope (%) | - | 1-3 | 0-1 | 0-3 |  |
| Water table depth (m) | - | >1.5 | >2.0 | >2 |  |
| Drainage Class | 0.0-1.2 | M.W.D. | W.D. | E.D. |  |

M.W.D., Moderately well drained; W.D., well drained; E.D., excessively drained; CEC, Cation exchange capacity; ^1^Fine-loamy, mixed, superactive, mesic Aquic Hapludolls loam, Lamberton Redwood County (44.237606, -95.307648); ^2^Fine-silty over sandy or sandy-skeletal, mixed, superactive, mesic Typic Hapludolls, at Rosemount Research and Outreach Center Rosemount, MN, Dakota County (44.715331, -93.099682); ^3^ Sandy, mixed, frigid Entic Hapludolls at Becker, Sherburne County, (45.345868, -93.853464). Note. Optimized θ_FC_ and θ_PWP_ (values in parentheses) were used for model calibration.

S2. Field trials data used for model calibration.

These include data collected at the Rosholt Research Farm, Westport, MN located in Rosholt Form, Pope County during the years 2011 to 2014 with continuous corn (C-C) and corn-soybean/soybean-corn (C-Sb) rotations (Rubin et al., 2016), and with rye planted in these rotations during and 2015-16-2016-17 (Ricks and Fernandez, 2019; Tahir et al., 2025). Data regarding alfalfa, corn and soybean were also collected from field trials (2010-2017) reported in Minnesota Agricultural Experiment Station and the College of Food, Agricultural and Natural Resource Sciences reports from Richmond (Stearns County), Rosemount (Dekota county), Lamberton (Redwood County) (available at <https://varietytrials.umn.edu/>), and Becker (Sherburne County) (<https://extension.umn.edu/forage-harvest-and-storage/seeding-year-harvest-management-alfalfa#sources-1048310>). Finally, corn crop EPIC auto-irrigation scheduling data were available for corn at the Becker and Westport sites from 2019-2021 (Singh et al., 2023). Model efficiency for rye crop was assessed based on only Rosholt farm data, and for EPIC irrigation scheduling reliable real data were available from the Rosholt farm and Sand Plain Research Farm (Becker site).

S3. Soil types, crop management, and irrigation scheduling

Estherville-Hawick complex, Sandburg, and Osakis soils at selected sites from the Bonanza Valley were classified as sandy, mixed, mesic/frigid -Typic/Calcic/Aquic Hapludolls. Estherville and Renshaw soils were fine-loamy over sandy mixed, superactive, frigid-Typic/-Calcic Hapludolls. Arvilla was coarse-loamy, mixed, superactive, frigid Calcic Hapludolls. Deeper depths (0.35-1.20 m) consisted of coarser texture, compared to the surface 0.35 m layer. Soils have slopes of 0-6%, moderately rapid permeability in the upper horizons to give somewhat excessive drainage, and rapid or very rapid permeability in the underlying parent material. Soil water contents at field capacity (θ_FC_) and permanent wilting point (θ_PWP_), cation exchange capacity, and SOM of these soils were generally low in the 0-35 cm depths, and further decreased at deeper depths. Surface 0-0.35 cm of Arvilla, Sandberg, Estherville-Hawick, Estherville, Osakis and Renshaw soils had 1.88, 1.50, 1.66, 2.89, 2.87, and 1.77% SOM, respectively. The six target soils accounted for 46.8, 4.5, 16.2, 3.7, 7.0, and 1.6% of the total (13,375 ha) irrigated area under these rotations.

Corn, soybean and alfalfa were planted at a population of 86,500, 445,000 and 2.5 million plants ha^-1^, respectively. Corn and soybean crop sowing dates varied from the last week of April to mid-May depending upon the weather conditions, while the harvesting date was during the last ten days of September. Alfalfa was planted during the first week of May and harvest occurred three times (on June 15, August 1, and September 15). Rye plant population was 0.8 and 1.5 million when planted after corn and soybean, respectively. Rye crop was killed by herbicide, and then plowed into the soil ten days prior to corn/soybean sowing.

Temporal distribution of the irrigation water with actual dates were not available, except at the Rosholt Farm (Arvilla soil) (Ricks and Fernandez, 2019; Singh et al., 2023). Irrigation distribution (amount and time) at each site was set by comparison with the Rosholt farm’s irrigation scheduling data, where water was applied using the checkbook method. The EPIC model was first calibrated for Arvilla soil, and then for all other five soil types with four replications under C-C, C-Sb/Sb-C and A-C/C-A rotations, using site specific irrigation water use (Figure S2), soil parameters (Table 1), calibration parameters for corn, soybean, alfalfa (Table S2), and irrigation water permit data (Figure S2). Irrigation depths applied at each site were estimated based on the amount of water applied as indicated by an irrigation water permit and dividing it by the area under the central pivot. EPIC was then run for C-C, C-Sb, and A-C crop rotations with rye as cover crop, where rye crop parameters were adjusted using rye crop field trial data (Ricks and Fernandez, 2019).

For EPIC auto-irrigation, minimum rate and interval of irrigation was adjusted to 10 mm and 5 days, respectively. The maximum rate of irrigation was adjusted to 25.4 mm. Adjustment of the matric potential at auto-trigger point was based on crop type and soil texture. Irrigation trigger in EPIC is based on soil moisture content of the upper 0.2 m soil layer. The matric potential trigger value was lowered to a level that did not generate a significant decrease in grain/forage yield of the crop. The maximum irrigation application rate for 5-day intervals was set to 25.4 mm, corresponding to the maximum ET_c_ rate observed this interval. A similar procedure was adopted by Wriedt et al. (2009) for auto-irrigation of different crops on a long-term basis.

A total of 322 irrigated sites (13,375 ha), including 113 (4881.1 ha) under C-C, 151 (6656.3 ha) under C-Sb/Sb-C, and 58 (1837.7 ha) under C-A/A-C were investigated using EPIC modeling (Figure S2). Sites with more than one soil type were simulated separately using soil specific physic-chemical properties. Area under each sprinkler irrigation system was known (Figure S2).

**TABLE S2.** EPIC calibrated values of plant growth and N uptake.

| Parameters | --------------Optimized values-------------- | | | |
| --- | --- | --- | --- | --- |
|  | Corn | Soybean | Alfalfa | Rye |
| WA, Biomass energy ratio | 43 | 25 | 20 | 38 (35) |
| DMLA, Maximum leaf area index | 5.5 | 5.1 | 6.2 | 2.0, 4.5* |
| H.I., Harvest Index | 0.52 (0.5) | 0.32 (0.3) | 0.001 | 0.001 |
| TBS, Minimum temp. for plant growth (°C) | 7 (8) | 9 (10) | 1 | 0 |
| TOP, Opt. temp. (C) for plant growth (°C) | 25 | 25 | 25 | 15 |
| DLAI, Growing season fraction when leaf area declines | 0.9 (0.8) | 0.9 (0.8) | 1 | 1 |
| BN_2_, N fraction at 50% maturity | 0.016 (0.0164 | 0.025 (0.027) | 0.026 (0.024) | 0.023 (0.021 |
| BN_3_, N fraction at maturity | 0.012 (0.0128) | 0.023 (0.025) | 0.022 | 0.012 (0.0125) |
| CNY, Fraction of nitrogen in yield | 0.015 (0.0175) | 0.055 (0.069) | 0.050 | 0.028 |
| RDMX, Maximum rooting depth (m) | 1.2 (2) | 1.2 (2) | 1.2 (2) | 1.2 (2) |

*, First and second values are used for rye planted in corn and soybean/alfalfa, respectively. Note. Values in parentheses donate to the default values.

**TABLE S3** EPIC model PARM value adjustments and input parameter settings for auto-irrigation.

| Parameters | Parameter description | Value |
| --- | --- | --- |
| PARM 5 | θ in top 0.5 m soil as fraction of W.P. | 0.3 |
| ˝ 20 | Microbial decay rate coefficient | 0.65 |
| ˝ 57 | N volatilization coefficient | 0.08 |
| ˝ 68 | N fixation upper limit | 10 |
| ˝ 75 | Runoff Adjustment factor | 0.7 |
| Auto-irrigation |  |  |
| IRR | Irrigation code (Sprinkler) | 1 |
| BIR | Irrigation trigger (kPa in 0.2 m soil layer) | -300, -450^*^ |
| EFI | Runoff fraction (-) | 0 |
| IRI | Minimum Irrigation interval (days) | 5 |
| ARMN | Minimum application rate (mm) | 10 |
| ARMX | Maximum application rate (mm) | 25.4 |
| IET | Potential ET equation | Penman-Monteith |

^*^1st and 2nd values were used for loamy coarse sand/sandy loam, and loam soils, respectively.

S4. EPIC model calibration detail

N contents in grain yield were important for corn and soybean crops. The optimized value of DMLA was 5.5, 5.1, and 6.2 for corn, soybean, and alfalfa, respectively. DMLA value for rye was 2.0 and 4.5 when seeded in corn and soybean, respectively. The maximum rooting depth considered was 1.2 m for all crops in order to estimate nitrate-N leaching losses at that depth. DMLA values for different crops were optimized to match METRIC-EEFlux ET_c_ values to EPIC model predictions to get required biomass/yield. Calibrated values of HI for EPIC model simulation of corn (0.52) and soybean (0.32) were slightly higher than default values. Alfalfa and rye crops were harvested before maturation, so very low values of HI were used. The optimized value of N contents at maturity (BN_3_) for corn (0.12), soybean (0.023), alfalfa (0.022), and rye (0.012) were slightly lower than default values. Rye was harvested at mid-stage, where N contents at 50% maturity (BN_2_) value (0.023) was slightly higher than default value (0.0215), however, lower than the field estimated values of about 0.028 (Ricks and Fernandez, 2019). Meta analysis of different locations in USA indicated that crops in sandy soils have lower response to applied N compared to finer texture soils (Tremblay et al., 2012). The EPIC model shows large reductions in yield if temperature falls below TBS (Minimum temp. for plant growth) during the crop growth period. However, crop varieties adapted to Minnesota’s local climate did not show such temperature stress. Thus, TBS values for corn and soybean were optimized 1 °C lower than the default values. Alfalfa and rye were harvested for forage yield at DLAI values of 1, indicating that there was no decrease in LAI before harvest. Rye seeds were broadcast during the final growth stages of corn/soybean crops, resulting in an overlap of about 15-20 days for rye seeding and harvest of the primary crop. EPIC is unable to simulate intercropping. To overcome this limitation of EPIC, corn/soybean DLAI was increased to 0.9 from a default value of 0.8. Moreover, harvest date of corn/soybean was adjusted ten days earlier than actual harvest, and rye growth was adjusted by increasing the biomass energy ratio from a default value of 0.35 to 0.38.

SSURGO data indicate (Table 1) that soil water retention and SOM decreased, while K_fs_ and bulk density increased at deeper depths, and for coarser texture soils, compared to medium texture soils. Soil water content at field capacity (θ_FC_) and wilting point (θ_WP_) were also optimized (Table 1) to get deep percolation and ET_c_ in close content to the measured values. The calibrated value of microbial decay-rate was lower (0.65) than the default value (1.0) to more accurately simulate net N mineralization rates from SOM under cool temperature conditions in Minnesota. The minimum value of soil water content in upper 0.5 m soil was optimized to a lower value of 0.3, compared to the default value (0.5). The runoff adjustment parameter was set higher (0.7) than the default value (0.0-0.3) to match field observations of very low or no runoff. A lower value (0.08) for the N volatilization coefficient was used to match the model output with literature values for the Midwestern corn belt. N fixation coefficient for soybean and alfalfa was set at a value of 10, lower than the default value (20), in order to better represent the coarse textured soils of Bonanza Valley.

**TABLE S4** Biomass and N uptake for rye planted after corn and soybean crop.

|  | Rye in C (C-C) | Rye in C (C-Sb) | Rye in C (A-C) | Rye in Sb (C-Sb) |
| --- | --- | --- | --- | --- |
| Biomass (t/ha) | 0.89 ± 0.31* | 0.99 ± 0.37 | 1.22 ± 0.33 | 2.08± 0.65 |
| N uptake (kg/ha) | 20.3 ± 4.5 | 22.7 ± 4.7 | 28.1 ± 4.9 | 47.8 ± 11.9 |

* Means± standard deviation


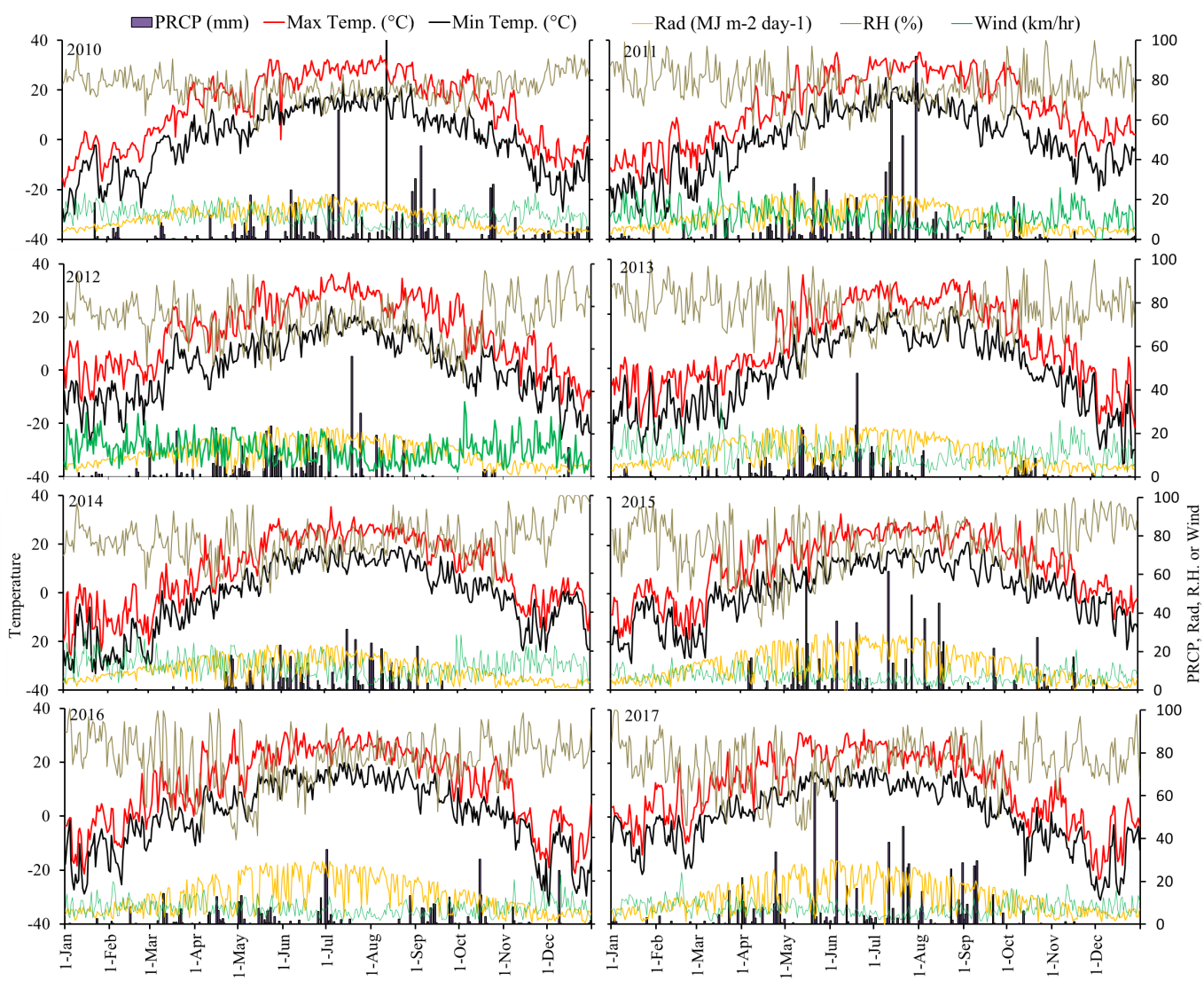


**FIGURE S1** Climatic data (2010-2017) used for EPIC modeling.


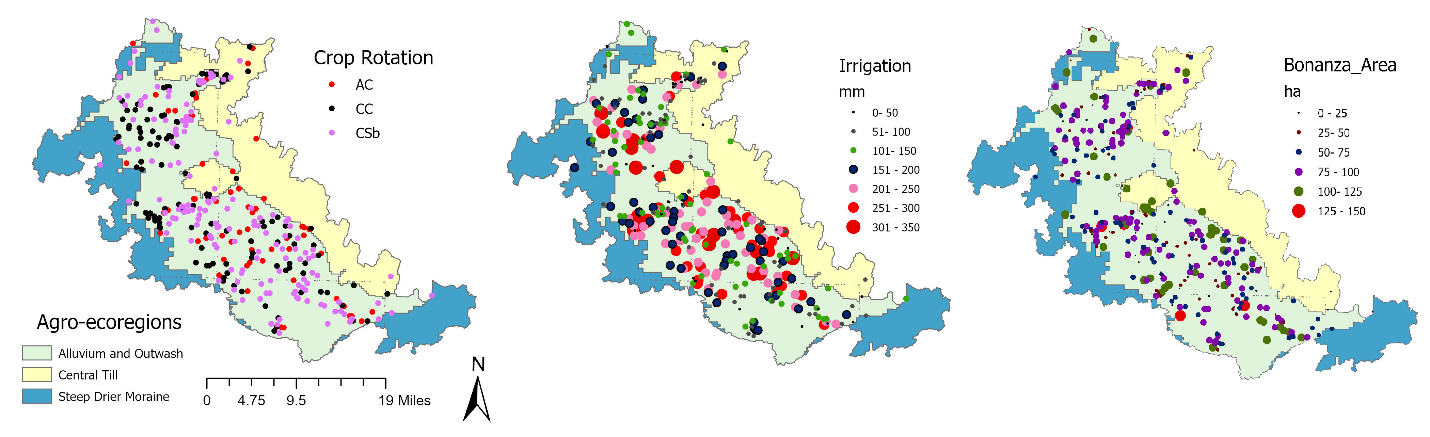


**FIGURE S2** Crop rotation, irrigation depth applied under water permits, and area under each central pivot system used for EPIC modeling assessment in the Bonanza Valley.

**
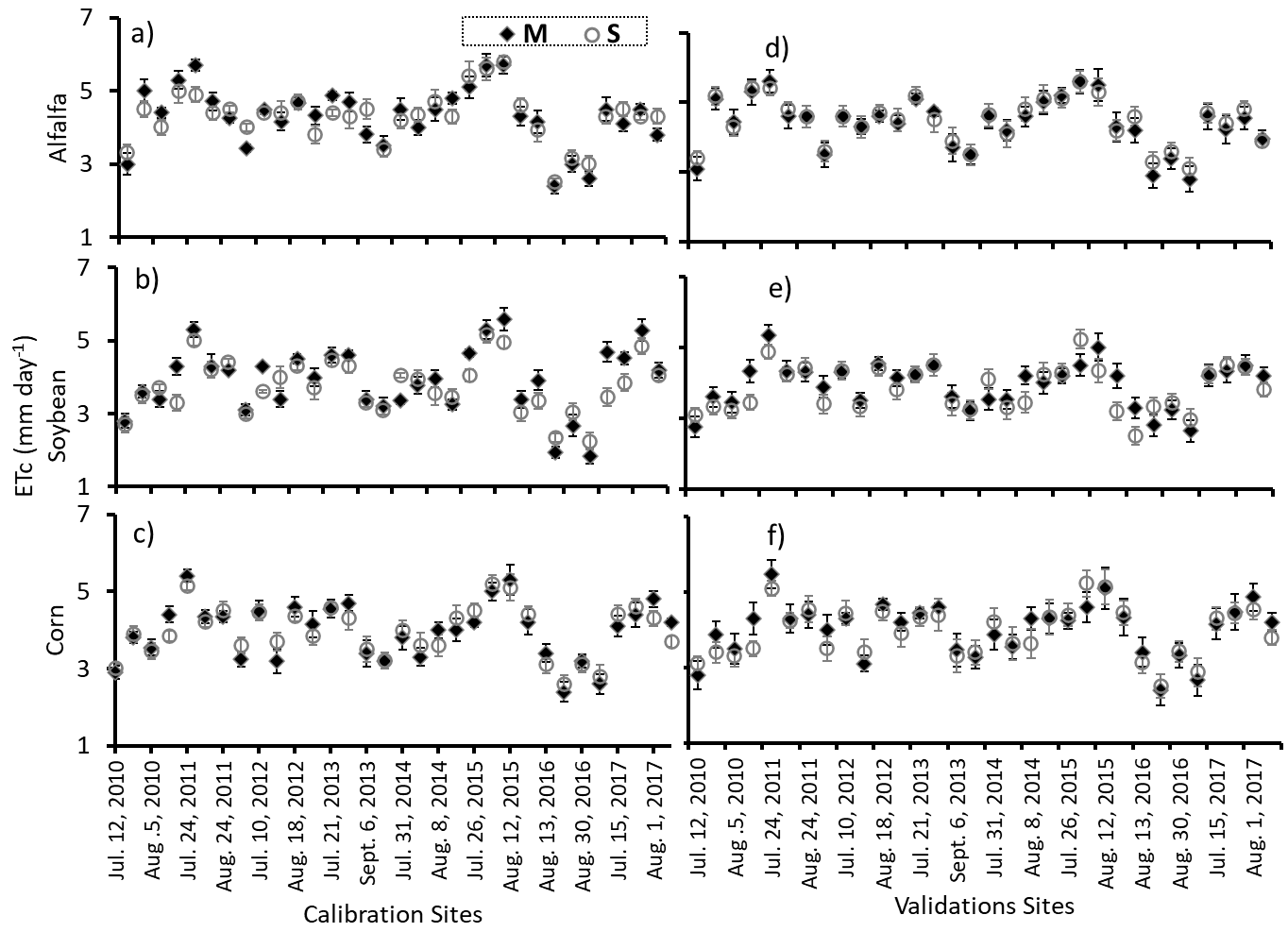
**

**FIGURE S3** METRIC-EEFlux measured (M) and EPIC simulated (S) ET_c_ values under different crops, for calibration and validation.

Note. Data represent the means of six soils with four replications.

**
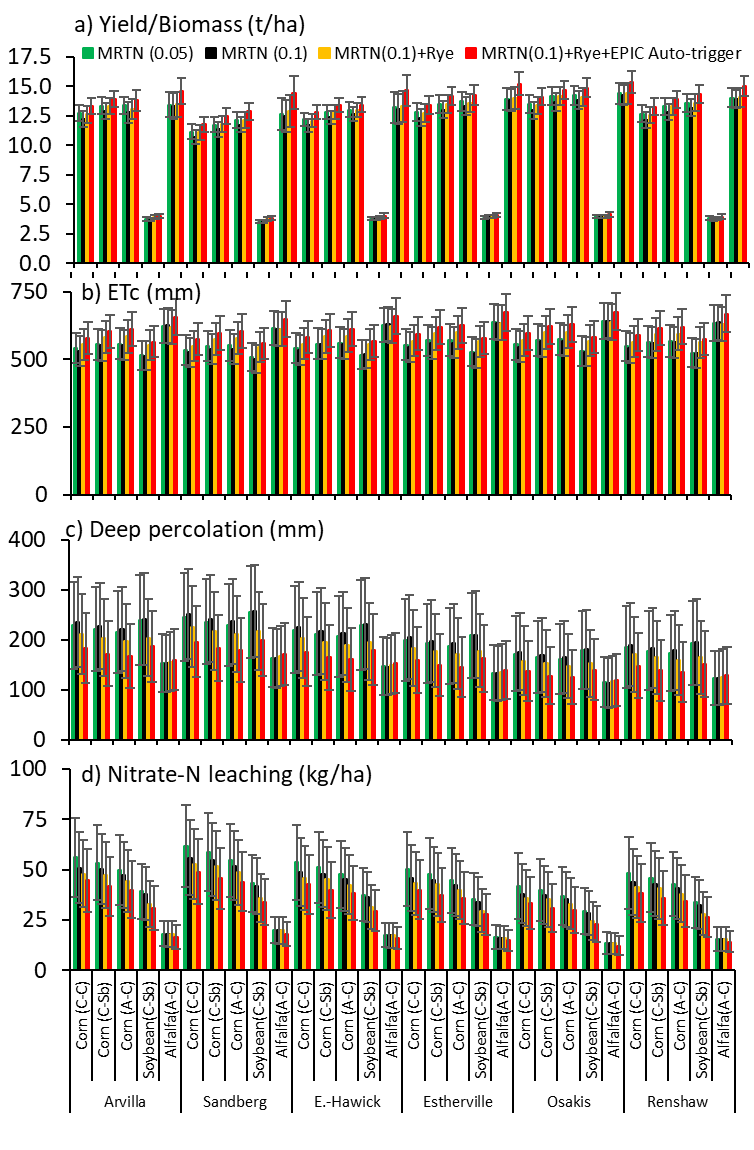
**

**FIGURE S4.** Crop yield/biomass, ET_c_, deep percolation, and nitrate-N leaching losses under different soil types.

**
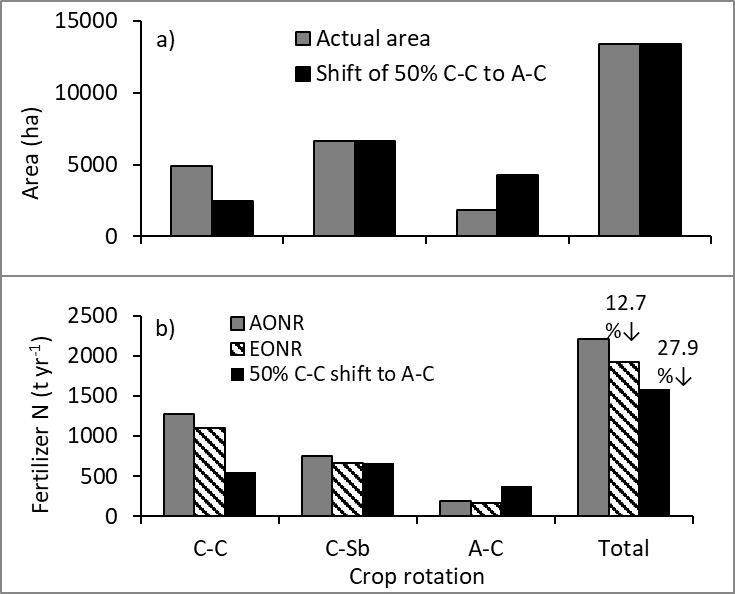
**

**FIGURE S5** Area and fertilizer N input under different crop rotations in Bonanza valley under alternative management scenarios.
